# Breast Cancer Incidence Trends in Older US Women by Race, Ethnicity, Geography, and Stage

**DOI:** 10.1001/jamanetworkopen.2025.16947

**Published:** 2025-06-24

**Authors:** Erica J. Lee Argov, Michelle L. Lui, Anita G. Karr, Parisa Tehranifar, Rebecca D. Kehm

**Affiliations:** 1 Department of Epidemiology, Mailman School of Public Health, Columbia University, New York, New York; 2 Herbert Irving Comprehensive Cancer Center, Columbia University Medical Center, New York, New York

## Abstract

**Importance:**

Breast cancer (BC) incidence trends are known to vary by race, ethnicity, and geography among younger women. Less is known about trends among older women (aged ≥65 years), who are typically aggregated, despite different screening guidelines for those older than 74 years.

**Objective:**

To disaggregate US BC incidence trends among older women (ages 65-74, 75-84, and ≥85 years) according to stage at diagnosis, race and ethnicity, hormone receptor subtype, and geography.

**Design, Setting, and Participants:**

This population-based cross-sectional study included women aged 65 years and older who were diagnosed with BC from January 2001 to December 2019. Data came from the US Cancer Statistics public use database, with information on women in all 50 US states. Data were analyzed from March to June 2024.

**Exposure:**

Age (65-74 years, 75-84 years, and ≥85 years).

**Main Outcomes and Measures:**

Age-adjusted BC incidence rates were obtained, and average annual percent changes (AAPCs) from 2001 to 2019 were estimated using joinpoint regression for each age group, stratified by stage at diagnosis, race and ethnicity, and geography.

**Results:**

From 2001 to 2019, 2 278 611 women (1 249 750 [54.9%] aged 65-74 years; 119 287 [5.2%] Hispanic [all races], 205 738 [9.0%] non-Hispanic Black, and 1 826 084 [80.1%] non-Hispanic White) were diagnosed with BC. Age-adjusted BC incidence rates were 530.4 (95% CI, 529.5 to 531.3) per 100 000 persons, 515.3 (95% CI, 514.2 to 516.5) per 100 000 persons, and 376.8 (95% CI, 375.3 to 378.2) per 100 000 persons for ages 65 to 74 years, 75 to 84 years, and 85 years and older, respectively. Annually, incidence rates increased in women aged 65 to 74 years (AAPC, 0.4%, 95% CI, 0.2% to 0.6%), remained stable in women aged 75 to 84 years, and decreased in women aged 85 years or older (AAPC, −1.1%, 95% CI, −1.4% to −0.8%). In women aged 65 to 74 years, the AAPC was 7 to 11 times higher in Hispanic, non-Hispanic American Indian or Alaska Native, non-Hispanic Asian or Pacific Islander, and non-Hispanic Black women compared with non-Hispanic White women. Across all age groups, regional stage diagnoses decreased (ages ≥65 years: AAPC, −0.8%, 95% CI, −1.1% to −0.5%), while distant stage diagnoses increased (ages ≥65 years: AAPC, 1.3%, 95% CI, 1.2% to 1.6%). Trends for in situ and localized diagnoses varied by age. Across all age groups, non-Hispanic Black women had the highest proportion of hormone receptor and *ERBB2*–negative cancers.

**Conclusions and Relevance:**

In this population-based cross-sectional analysis of BC incidence trends among older US women, racial and ethnic as well as stage-specific patterns differed across age groups, highlighting the importance of disaggregating BC incidence rates into age groups better aligned with screening guidelines. Future research is needed to directly examine the contribution of screening patterns to these trends and their impact on BC mortality.

## Key Points

**Question:**

Do breast cancer incidence trends differ across disaggregated age groups among older women, particularly those affected by reduced screening guidelines?

**Findings:**

In this cross-sectional study of 2 278 611 women diagnosed with breast cancer from 2001 to 2019, breast cancer incidence trends differed among the oldest women (aged ≥85 years) compared with younger women (aged 65-74 years), particularly by race and ethnicity and stage at diagnosis. Non-Hispanic Black women continued to have the highest proportion of triple-negative breast cancers across all age groups.

**Meaning:**

The findings of this study suggest that breast cancer incidence trends among older women should be disaggregated by age groups to account for age-related differences in screening and other risk factors.

## Teaser

This cross-sectional study disaggregates trends in breast cancer incidence among women aged 65 years, who are affected by different screening guidelines based on age.

## Introduction

Breast cancer (BC) is the most common non–skin cancer malignant neoplasm and the second leading cause of cancer-related death in women in the United States.^1^ BC incidence rates have fluctuated over time, influenced by shifts in population-level screening practices and risk factor distributions. For example, the rapid adoption of mammography in the 1980s led to increased tumor detection, while the decline in hormone therapy use for menopause following the 2002 Women’s Health Initiative randomized clinical trial contributed to later decreases in incidence.^2^ BC incidence has increased over time in women younger than 40 years, with invasive BC incidence rates increasing by 0.5% per year between 2004 and 2019 and distant stage disease increasing by 3.5% annually in this age group.^3^ These trends vary by different demographic factors, such as race, ethnicity, and geography.^3,4^ For example, distant stage disease increased by 3.5% per year from 1992 to 2009 in non-Hispanic Black women younger than 40 years compared with 1.9% per year in Hispanic women younger than 40 years.^3^

While BC incidence trends in younger US women have received significant attention, few studies closely examine trends in the oldest women—a notable gap, as women aged 65 years and older are one of the fastest-growing groups in the United States, representing 16.8% of the population in 2020, and have the highest BC incidence rates, which peak around age 70 years for all racial and ethnic groups.^2,5^ The magnitude of this peak ranges from approximately 325 per 100 000 persons for non-Hispanic Asian American and Pacific Islander women to around 500 per 100 000 persons for non-Hispanic White women.^2^ Many studies exclude women aged 85 years and older from trend analyses and often aggregate women aged 70 to 84 years into a single group, overlooking differences in mammography recommendations within this age range.^6,7^ Due to insufficient data on the balance of benefits and harms of mammography in older women, as well as the lower life expectancy and higher risk of comorbidities associated with aging, most professional organizations do not recommend routine screening after age 74 years. The US Preventive Services Task Force (USPSTF) does not make a recommendation for or against mammography screening in women older than 74 years, the American College of Physicians recommends mammography cessation in average-risk women after age 74 years, and other organizations recommend tailoring decisions on functional health, comorbidities, and life expectancy.^7,8,9,10^ Given that screening mammography participation influences incidence rates and the distribution of stage at diagnosis, it is essential to examine trends over time across screening-relevant age groups to determine whether observed trends align with changes in mammography participation or indicate other contributing factors to BC incidence in older women.

This study leverages cancer registry data from all 50 US states to examine BC incidence rates among women aged 65 years and older. To account for different screening guidelines, we used 10-year age groups and included women older than 85 years. We examined BC incidence trends within these age groups, stratified by race and ethnicity, geography, and stage at diagnosis. Additionally, we examined age-specific incidence rates by molecular subtypes to investigate variations in aggressive tumor features among older women.

## Methods

### Data Source and Study Population

We used the US Cancer Statistics (USCS) public use database to analyze trends in BC incidence rates from 2001 to 2019 among US women aged 65 years and older.^11^ The USCS is a comprehensive, population-based cancer surveillance system covering approximately 99% of the US population, combining data from the Surveillance, Epidemiology, and End Results (SEER) program and the Centers for Disease Control and Prevention (CDC) National Program of Cancer Registries (NPCR).^11^ All new cancer diagnoses from patient records at medical facilities (eg, hospitals, clinics, radiation or surgical facilities, pathology laboratories) are reported to central cancer registries using uniform coding and combined annually.^11^ Patient demographic data (eg, race and ethnicity) and medical information (eg, stage at diagnosis) are reported to cancer registries using medical or administrative records, including death certificates.^11^ We received approval to use deidentified cancer incidence data through a USCS Public Use Research Data Agreement with the CDC and the National Cancer Institute (NCI). The study was exempt from ethical review and the requirement for informed consent and complies with Strengthening the Reporting of Observational Studies in Epidemiology (STROBE) reporting guidelines.^12^

We used SEER*Stat software^13^ to obtain annual incidence rates age adjusted to the 2000 US standard population based on 19 age groups (Census P25-1130)^14^ and 95% CIs for in situ and invasive BC cases diagnosed from January 1, 2001, through December 31, 2019, in females aged 65 years or older. Analyses were conducted in 2024 using the 2023 NPCR/SEER submission^15^ which contains diagnoses through 2021. We excluded diagnoses after 2019 due to significant disruptions in cancer diagnoses caused by the COVID-19 pandemic.^16^ We also excluded male BC cases due to sparse data when stratified by diagnosis age and other characteristics (BC incidence hereafter refers to female BC incidence). For each age group at diagnosis (65-74 years, 75-84 years, and ≥85 years as well as the overall sample [≥65 years]), we obtained BC incidence rates overall and stratified by race and ethnicity, metropolitan status, geographic region, and stage at diagnosis.

Race and ethnicity were categorized as Hispanic, non-Hispanic American Indian or Alaska Native (hereafter, American Indian or Alaska Native), non-Hispanic Asian or Pacific Islander (hereafter, Asian or Pacific Islander), non-Hispanic Black (hereafter, Black) and non-Hispanic White (hereafter, White). Metropolitan status (metropolitan, nonmetropolitan, and unknown) was based on Rural-Urban Continuum Codes from the US Department of Agriculture.^17^ Geographic region (Midwest, Northeast, South, and West) was designated by NPCR based on the US Census. We defined stage at diagnosis as localized, regional, distant, and unknown by creating a user-defined variable within SEER*Stat using the Combined Summary Stage variable (SEER combined summary stage 2000 [2004-2017] and derived summary stage 2018 [2018+]) that combined all forms of regional staged BC. Unknown stage includes unknown, unstaged, unspecified, and cases identified only from the death certificate, where stage is not included (94 828 diagnoses [4.2% of diagnoses among those aged ≥65 years]).

We used SEER’s breast subtype variable, only available for diagnoses from 2011 to 2019, which groups tumors into 4 categories based on joint expressions of hormone receptors (HR) capturing estrogen and progesterone receptors and with or without the *ERBB2* (formerly *HER2*) marker.^18^ Molecular subtypes include luminal A, with HR expression and without *ERBB2* expression; luminal B, with HR and *ERBB2* expressions; *ERBB2*-enriched, without HR expression and with *ERBB2* expression; triple negative, without HR or *ERBB2* expressions; and unknown.^19^ The unknown category included cases where samples had been sent out for testing but no results were reported or where values were unknown or blank.

### Statistical Analysis

We used SEER*Stat to calculate incidence rate ratios and 95% CIs, applying the modification option for confidence interval estimation from Tiwari et al^20^ to compare age-specific BC incidence rates from 2001 to 2019 across racial and ethnic groups, geographic areas, and stages at diagnosis. We descriptively compared the proportion each BC molecular subtype (luminal A, luminal B, *ERBB2*-enriched, and triple negative) that contributed to the total incidence rate from 2011 to 2014 and 2015 to 2019, stratified by age group, race and ethnicity, geographic region, and stage at diagnosis.

We calculated the average annual percent change (AAPC) in BC incidence rates from 2001 to 2019 using joinpoint regression (Joinpoint software version 5.0 [released April 2023] from the NCI), which fits a series of joined straight lines on a logarithmic scale to the trends in the annual age-standardized rates.^21,22^ Models allowed for up to 3 joinpoints and required a minimum of 4 observations between adjacent inflection points. We used the weighted bayesian information criterion method to select the best fitting log-linear model.^23^ The AAPC was calculated as a weighted average of the annual percent change (APC) values, where each APC represents the slope of the joinpoint regression line within a given segment. The weights were proportional to the length of each segment within the specified interval. To determine whether the AAPC was statistically different from zero (*P* < .05), a 2-sided *t* test was used for 0 joinpoints, and a 2-sided *z* test was used for 1 or more joinpoints. APCs were calculated using the same approach for each segment between inflection points. Rates were considered to increase or decrease if *P* < .05; otherwise, rates were considered stable between 2001 to 2019 if the AAPC was not statistically significant at *P* < .05.

## Results

**Figure 1.**
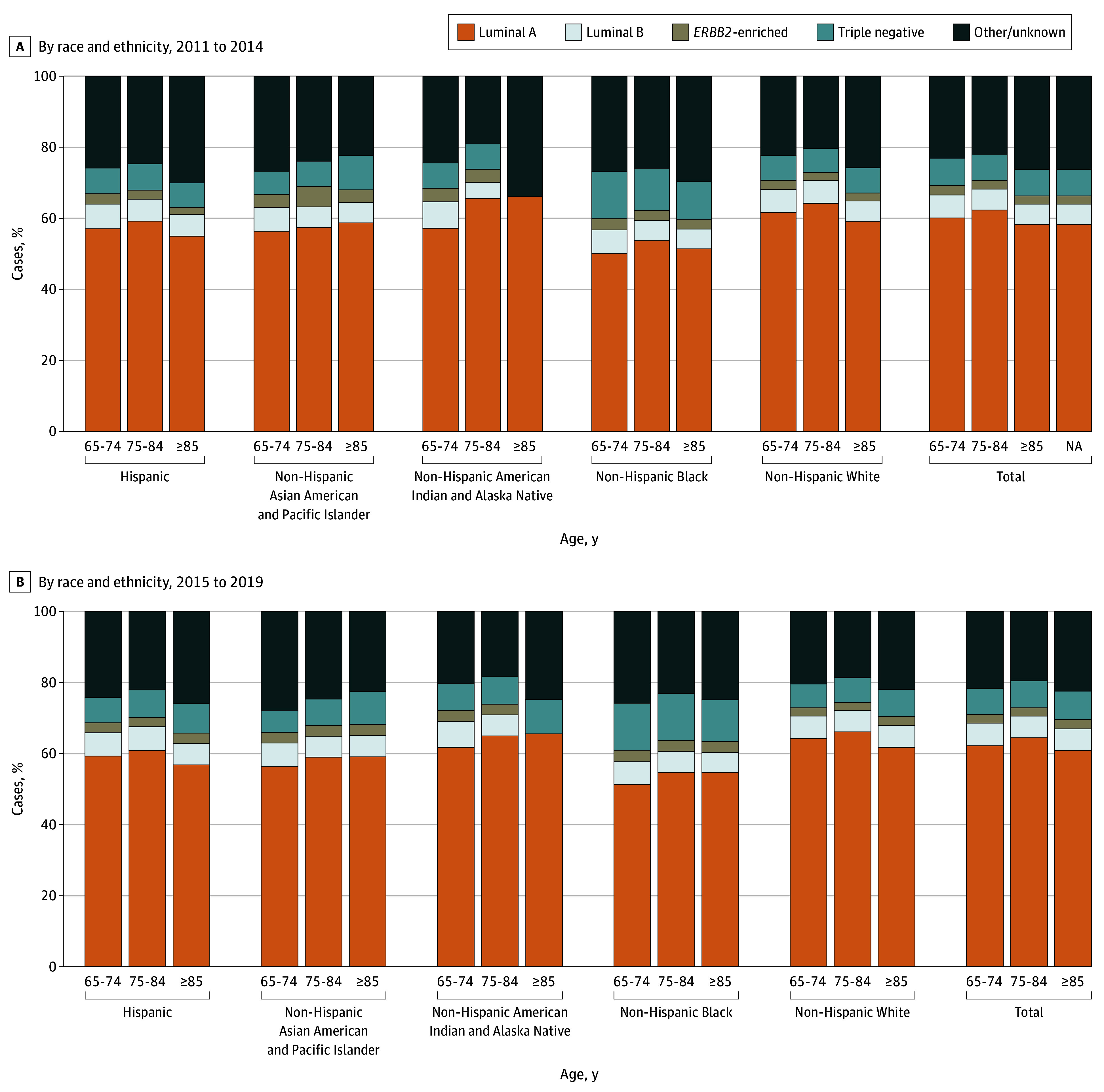
Molecular Subtype Composition of Age-Adjusted Invasive and In Situ Age-Adjusted Breast Cancer Incidence Luminal A is hormone receptor (HR)-positive and *ERBB2*-negative disease; luminal B, HR- and *ERBB2*-positive disease; *ERBB2*-enriched, HR-negative and *ERBB2*-positive disease; triple negative, HR- and *ERBB2*-negative disease. Some subgroups within bars had small cell sizes that required suppression (panel A, non-Hispanic American Indian or Alaska Native women with luminal B, *ERBB2*-enriched, and triple-negative disease; panel B, non-Hispanic American Indian or Alaska Native women with luminal B and *ERBB2*-enriched disease) and are not displayed.

From 2001 to 2019, a total of 2 278 611 (1 249 750 [54.9%] aged 65-74 years; 119 287 [5.2%] Hispanic [all races], 205 738 [9.0%] non-Hispanic Black, and 1 826 084 [80.1%] non-Hispanic White) women were diagnosed with invasive or in situ BC. The overall age-adjusted BC incidence rate was 530.4 (95% CI, 529.5, 531.3) per 100 000 persons in those aged 65 to 74 years, 515.3 (95% CI, 514.2, 516.5) per 100 000 persons in those aged 75 to 84 years, and 376.8 (95% CI, 375.3, 378.2) per 100 000 persons in those aged 85 years and older (Table 1). The distribution of molecular subtypes was similar across age groups, with luminal A cancers constituting more than half of all BC cases in each group (Figure 1; eTable 1 in Supplement 1). The distribution of molecular subtypes remained relatively consistent within each age group between 2011 to 2014 and 2015 to 2019. In both periods, Black women had higher rates of triple-negative BC across all age groups compared with women belonging to the other racial and ethnic groups (eTable 1 in Supplement 1).

**Table 1.** Age-Adjusted Breast Cancer Incidence Rates and Rate Ratios Among Older Women, United States Cancer Statistics Database, 2001-2019

Subgroup	All ages (≥65 y)		65-74 y		75-84 y		≥85 y	
	Rate (95% CI)^a^	Incidence rate ratio (95% CI)	Rate (95% CI)^a^	Incidence rate ratio (95% CI)	Rate (95% CI)^a^	Incidence rate ratio (95% CI)	Rate (95% CI)^a^	Incidence rate ratio (95% CI)
Overall	506.2 (505.5-506.8)	NA	530.4 (529.5-531.3)	NA	515.3 (514.2-516.5)	NA	376.8 (375.3-378.2)	NA
Race and ethnicity								
Hispanic (all races)	367.1 (365.0-369.2)	0.70 (0.69-0.70)	393.5 (390.6-396.4)	0.71 (0.71-0.72)	362.0 (358.2-365.7)	0.67 (0.67-0.68)	269.7 (264.4-275.1)	0.70 (0.68-0.71)
Non-Hispanic American Indian or Alaska Native	377.3 (369.1-385.5)	0.72 (0.70-0.73)	401.5 (390.8-412.4)	0.73 (0.71-0.75)	381.2 (366.2-396.7)	0.71 (0.68-0.74)	262.6 (242.0-284.4)	0.68 (0.63-0.74)
Non-Hispanic Asian or Pacific Islander	327.5 (324.8-330.2)	0.62 (0.62-0.63)	371.8 (368.0-375.6)	0.67 (0.67-0.68)	304.5 (299.8-309.3)	0.57 (0.56-0.58)	205.4 (199.2-211.9)	0.53 (0.51-0.55)
Non-Hispanic Black	491.6 (489.5-493.8)	0.93 (0.93-0.94)	516.1 (513.1-519.0)	0.93 (0.93-0.94)	490.1 (486.2-493.9)	0.91 (0.90-0.92)	392.0 (386.7-397.3)	1.01 (1.00-1.03)
Non-Hispanic White	526.9 (526.1-527.7)	1 [Reference]	552.2 (551.1-553.3)	1 [Reference]	538.1 (536.7-539.4)	1 [Reference]	386.8 (385.2-388.4)	1 [Reference]
Region								
Midwest	517.6 (516.2-519.0)	0.97 (0.97-0.98)	539.8 (537.8-541.9)	0.97 (0.96-0.97)	530.3 (527.9-532.8)	0.97 (0.97-0.98)	386.4 (383.4-389.3)	1.01 (1.00-1.02)
Northeast	531.1 (529.6-532.7)	1 [Reference]	556.9 (554.7-559.1)	1 [Reference]	544.5 (541.8-547.2)	1 [Reference]	382.8 (379.7-385.9)	1 [Reference]
South	487.2 (486.1-488.3)	0.92 (0.91-0.92)	512.2 (510.7-513.7)	0.92 (0.92-0.92)	492.8 (491.0-494.7)	0.91 (0.90-0.91)	364.6 (362.2-367.1)	0.95 (0.94-0.96)
West	504.5 (503.0-505.9)	0.95 (0.95-0.95)	529.9 (527.9-532.0)	0.95 (0.95-0.96)	510.4 (507.9-512.9)	0.94 (0.93-0.94)	379.0 (375.8-382.2)	0.99 (0.98-1.00)
Metropolitan status								
Metropolitan	514.1 (513.4-514.9)	1 [Reference]	539.2 (538.1-540.2)	1 [Reference]	523.7 (522.4-525.0)	1 [Reference]	380.0 (378.4-381.6)	1 [Reference]
Nonmetropolitan	467.7 (466.2-469.2)	0.91 (0.91-0.91)	487.6 (485.4-489.7)	0.90 (0.90-0.91)	475.3 (472.6-477.9)	0.91 (0.90-0.91)	361.1 (357.7-364.4)	0.95 (0.94-0.96)
Stage at diagnosis								
In situ	82.8 (82.6-83.1)	1 [Reference]	99.2 (98.8-99.6)	1 [Reference]	77.2 (76.7-77.6)	1 [Reference]	29.4 (29.0-29.8)	1 [Reference]
Localized	283.0 (282.5-283.5)	3.42 (3.40-3.43)	295.2 (294.5-295.9)	2.97 (2.96-2.99)	294.5 (293.6-295.3)	3.82 (3.79-3.84)	198.0 (197.0-199.1)	6.73 (6.64-6.83)
Regional	96.8 (96.5-97.1)	1.17 (1.16-1.17)	100.7 (100.3-101.2)	1.02 (1.01-1.02)	98.0 (97.5-98.5)	1.27 (1.26-1.28)	76.6 (76.0-77.3)	2.61 (2.56-2.65)
Distant	23.6 (23.5-23.8)	0.29 (0.28-0.29)	22.4 (22.2-22.6)	0.23 (0.22-0.23)	25.4 (25.1-25.6)	0.33 (0.33-0.33)	24.1 (23.7-24.4)	0.82 (0.80-0.83)
Unknown^b^	19.9 (19.8-20.0)	0.24 (0.24-0.24)	12.9 (12.7-13.0)	0.13 (0.13-0.13)	20.3 (20.0-20.5)	0.26 (0.26-0.27)	48.7 (48.1-49.2)	1.65 (1.63-1.68)

Abbreviation: NA, not applicable.

^a^ Rates include both in situ and invasive breast cancers. Rates are per 100 000 people and age-adjusted to the 2000 US standard population (19 age groups [Census P25-1130]).

^b^ Includes unstaged and death certificate–only cases.

**Figure 2.**
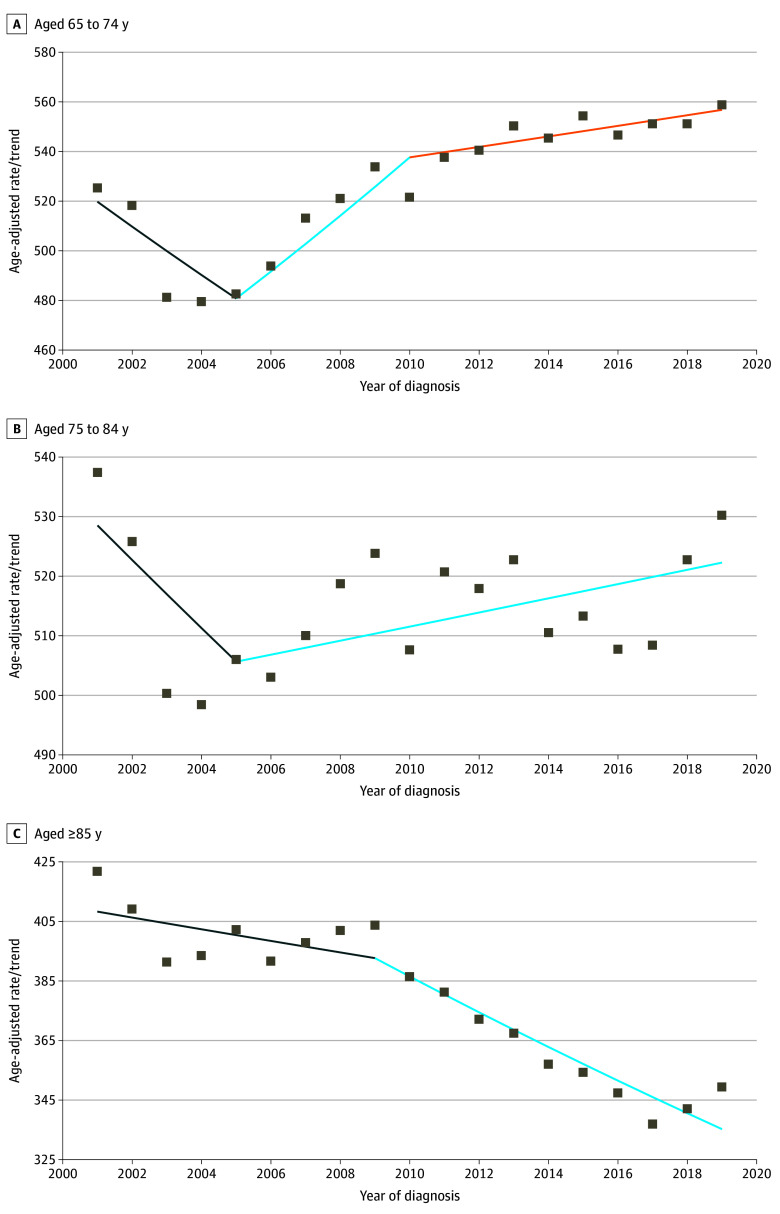
Overall Invasive and In Situ Breast Cancer Segmented Trends From 2001 to 2019 Rates are per 100 000 persons and age adjusted to the 2000 US Standard Population (19 age groups [Census P25-1130]). Figures represent the best fitting model using joinpoint regression allowing for a maximum of 3 joinpoints and requiring a minimum of 4 years within each segment and between each segment and either end. Due to COVID-19–related declines in incidence affecting the trend, 2020 was excluded from the joinpoint regression trendline and calculations, although it remains plotted.

From 2001 to 2019, BC incidence increased by 0.4% (95% CI, 0.2% to 0.6%) per year among those aged 65 to 74 years (Table 2). During this same period, incidence rates did not statistically significantly change for those aged 75 to 84 years and decreased by 1.1% (95% CI, −1.4% to −0.8%) per year in women aged 85 years and older. Figure 2 displays the segmented trends and APCs from 2001 to 2019, showing similar overall trends in women younger than 85 years (eTable 2 and eFigure in Supplement 1).

**Table 2.** AAPCs in Invasive and In Situ Breast Cancer Incidence Rates From 2001 to 2019 by Geographic and Patient Characteristics

Characteristic	AAPC (95% CI), %			
	All ages (≥65 y)	65-74 y	75-84 y	≥85 y
Total US	0.06 (−0.15 to 0.32)	0.38 (0.21 to 0.60)	−0.07 (−0.23 to 0.02)	−1.09 (−1.36 to −0.82)
Race and ethnicity				
Hispanic	0.57 (0.34 to 0.85)	1.36 (1.16 to 1.59)	−0.16 (−0.53 to 0.27)	−1.33 (−1.87 to −0.67)
Non-Hispanic American Indian or Alaska Native	0.90 (0.24 to 1.80)	1.77 (0.81 to 3.30)	0.08 (−0.60 to 1.11)	−0.36 (−2.31 to 2.71)
Non-Hispanic Asian or Pacific Islander	1.54 (1.17 to 2.01)	2.19 (1.85 to 2.66)	0.89 (0.52 to 1.34)	−0.71 (−1.61 to 0.47)
Non-Hispanic Black	1.21 (0.94 to 1.54)	1.62 (1.42 to 1.87)	1.04 (0.74 to 1.41)	−0.27 (−0.66 to 0.15)
Non-Hispanic White	0.00 (−0.19 to 0.20)	0.22 (0.04 to 0.45)	−0.01 (−0.17 to 0.27)	−1.04 (−1.36 to −0.73)
Region				
Midwest	0.21 (0.05 to 0.50)	0.42 (0.22 to 0.68)	0.09 (−0.05 to 0.30)	−1.50 (−1.82 to −1.17)
Northeast	0.43 (0.23 to 0.65)	0.75 (0.49 to 1.11)	0.25 (0.01 to 0.48)	−1.21 (−1.54 to −0.88)
South	0.21 (0.03 to 0.42)	0.51 (0.30 to 0.80)	0.03 (−0.15 to 0.32)	−0.66 (−0.93 to −0.33)
West	−0.42 (−0.69 to −0.08)	−0.08 (−0.29 to 0.17)	−0.63 (−0.84 to −0.29)	−1.15 (−1.59 to −0.69)
Metropolitan status				
Metropolitan	0.03 (−0.18 to 0.27)	0.35 (0.17 to 0.57)	0.02 (−0.27 to 0.31)	−1.06 (−1.37 to −0.73)
Nonmetropolitan	0.22 (0.07 to 0.46)	0.58 (0.39 to 0.93)	0.03 (−0.12 to 0.29)	−1.32 (−1.71 to −0.94)
Stage at diagnosis				
In situ	0.22 (−0.11 to 0.62)	0.66 (0.27 to 1.16)	−0.28 (−0.65 to 0.13)	−1.87 (−2.41 to −1.40)
Localized	0.57 (0.41 to 0.77)	0.95 (0.78 to 1.18)	0.38 (0.20 to 0.74)	−0.66 (−0.94 to −0.36)
Regional	−0.83 (−1.08 to −0.53)	−0.80 (−1.07 to −0.48)	−0.92 (−1.23 to −0.60)	−0.89 (−1.23 to −0.53)
Distant	1.33 (1.15 to 1.56)	1.13 (0.92 to 1.34)	1.64 (1.33 to 1.96)	1.64 (1.36 to 1.95)
Unknown^a^	−3.90 (−4.48 to −3.04)	−3.99 (−4.61 to −3.34)	−3.64 (−4.37 to −3.07)	−3.80 (−4.32 to −3.11)

Abbreviation: AAPC, average annual percentage change.

^a^ Includes unstaged and death certificate–only cases.

When stratified by race and ethnicity, White women had the highest BC incidence from 2001 to 2019, overall and in the 2 younger age groups, while Black women had the highest BC incidence among those aged 85 years and older. In those aged 65 to 74 years, BC incidence increased from 2001 to 2019 across all racial and ethnic groups, although at a much higher annual rate in Black (AAPC, 1.6%; 95% CI, 1.4% to 1.9%), American Indian or Alaska Native (AAPC, 1.8%, 95% CI, 0.8% to 3.3%), Asian or Pacific Islander (AAPC, 2.2%; 95% CI, 1.9% to 2.7%), and Hispanic (AAPC, 1.4%, 95% CI, 1.2% to 1.6%) women compared with White women (AAPC, 0.2%; 95% CI, 0.04% to 0.5%). In those aged 75 to 84 years, BC incidence increased in Black and Asian or Pacific Islander women (Black women: AAPC, 1.0%; 95% CI, 0.7% to 1.4%; Asian or Pacific Islander women: AAPC, 0.9%; 95% CI, 0.5% to 1.3%) and showed no statistically significant increases or decreases in the other racial and ethnic groups. In those aged 85 years and older, BC incidence decreased in White and Hispanic women (White women: AAPC, −1.0%; 95% CI, −1.4% to −0.7%; Hispanic women: AAPC, −1.3%, 95% CI, −1.9% to −0.7%), and did not statistically significantly change in the other racial and ethnic groups. eTable 2 and eFigure in Supplement 1 report the segmented trends further stratified by race and ethnicity.

When stratified by geographic region, the Northeast had the highest incidence of BC from 2001 to 2019 in those aged 65 to 74 years and 75 to 84 years, while the Midwest had the highest incidence in those aged 85 years and older. In those aged 65 to 74 years, BC incidence increased from 2001 to 2019 in all US regions except the West. In those aged 75 to 84 years, BC incidence increased in the Northeast (AAPC, 0.3%; 95% CI, 0.0% to 0.5%), decreased in the West (AAPC, −0.6%; 95% CI, −0.8% to −0.3%), and did not significantly change in the Midwest and South. In those aged 85 years and older, BC incidence decreased across all US regions, with annual decreases ranging from 0.7% (95% CI, −0.9% to −0.3%) in the South to 1.5% (95% CI, −1.8% to −1.2%) in the Midwest.

When stratified by metropolitan designation, BC incidence rates were higher in metropolitan areas compared with nonmetropolitan areas in all age groups. However, BC incidence trends were similar in metropolitan and nonmetropolitan areas from 2001 to 2019.

When stratified by stage at diagnosis, distant stage disease increased from 2001 to 2019 across all age groups (all women aged ≥65 years: AAPC, 1.3%, 95% CI, 1.2% to 1.6%), while regional and unknown stage disease decreased across all age groups. In situ BC incidence increased from 2001 to 2019 in women aged 65 to 74 years (AAPC, 0.7%; 95% CI, 0.3% to 1.2%), did not significantly change in women aged 75 to 84 years, and decreased in those aged 85 years and older (AAPC, −1.9%; 95% CI, −2.4% to −1.4%). Women aged 65 to 74 years had a nearly 3-fold higher incidence of in situ BC compared with those aged 85 years and older during 2001 to 2019.

## Discussion

This study provides an in-depth examination of recent BC incidence patterns in older women in the United States, a growing population with a high burden of BC. We evaluated recent BC incidence trends stratified by age groups that align with current screening guidelines, which recommend reduced screening for women older than 75 years.^8,24^ Notably, we distinguished trends among women aged 85 years and older, a group often excluded from BC incidence studies or combined with younger age categories, to provide a more comprehensive understanding of this population. Our analysis revealed variations in BC incidence rates from 2001 to 2019 across distinct age groups of older women, differences potentially overlooked in studies with broader age classifications.

Overall, we found that BC incidence rates were highest in those aged 65 to 74 years, which also had a higher rate of in situ disease and a lower rate of distant stage disease compared with the 2 older age groups. Interestingly, the distribution of BC molecular subtypes was similar across the 3 older age groups, suggesting that the observed stage-specific trends are unlikely to be attributed to variations in tumor biology. Instead, these stage-specific patterns may suggest a lower frequency of screen-detected, early-stage tumors in the older 2 age groups. We were unable to test this hypothesis in our study, given that the USCS database does not provide screening data. However, a recent study examining screening data before and after the 2009 USPSTF guideline change (no longer recommending biennial screening for those aged 75 years and older) identified reduced screening rates among women aged 75 years and older compared with women younger than 75 years, suggesting that guideline changes affect screening rates and thereby potentially incidence in these age groups.^25^ Further research should continue to investigate whether variations in screening practices contribute to the higher rates of advanced-stage disease in women aged 75 years and older.

When we examined BC incidence trends over time (2001-2019), we again identified age-specific differences among women aged 65 years and older. During this period, overall BC incidence increased in those aged 65 to 74 years, remained stable in those aged 75 to 84 years, and decreased by more than 1% annually in those aged 85 years or older. These age-specific BC incidence trends were mostly consistent across geographic designations (by region and metropolitan status); although the West was the only region where rates either decreased or remained stable across all 3 age groups. Interestingly, our recent research on geographic differences in BC incidence trends among younger women found that the West had the highest rate of increase for BC incidence in women aged 25 to 39 years.^4^ Together, these findings highlight the importance of disaggregating incidence trends and accounting for the intersection of age, geography, and other demographic factors when analyzing breast cancer patterns.

We identified notable differences in age-specific BC incidence trends when stratified by race and ethnicity. Specifically, White women were the only racial and ethnic group in which BC incidence did not significantly increase from 2001 to 2019 overall (all ages ≥65 years). Furthermore, while BC incidence increased in women aged 65 to 74 years across all racial and ethnic groups, the rate of increase was 7 to 11 times lower among White women compared with other groups. Black and Asian or Pacific Islander women were the only racial and ethnic groups in which BC incidence increased in those aged 75 to 84 years; and, these groups, along with American Indian or Alaskan Native women, did not experience a decrease in incidence in those aged 85 years or older. These patterns may reflect the increasing uptake of screening in many racially and ethnically minoritized groups,^26^ and studies describe unchanged screening rates among Black women aged 75 years and older after the USPTSF guideline change.^25^ However, the consistently higher prevalence of triple-negative BC in Black women compared with other racial and ethnic groups, even in the oldest age group, suggests that factors beyond screening may contribute to the higher burden of less treatable and more aggressive tumors in this group. The higher incidence of triple-negative BC in older Black women, which has also been observed in younger women,^27^ highlights the need to investigate factors that may contribute to racial and ethnic differences in BC incidence rates across the life course.

### Limitations

While this study’s strengths include the disaggregation of older age groups, the comprehensive inclusion of multiple stratifying factors, and the use of nationwide cancer registry data, our interpretations of stage and molecular subtype trends are limited by differences in the proportion of unknown stage and subtype by race and ethnicity and age, and changes in the proportion of unknown classifications over time.^28^ Additionally, molecular subtype data were only available from 2011 to 2019, limiting our ability to conduct extensive trend analyses for these variables. Furthermore, we were limited by the lack of data on corresponding screening rates by race and ethnicity, geography, stage at diagnosis, and subtype within which to situate these findings. We were also unable to account for changes in other known or emerging BC risk factors in our analysis. Nevertheless, this study is descriptive and intended to raise key avenues for future research, such as the contribution of screening and risk factors to BC incidence among the oldest women.

## Conclusions

In this population-based cross-sectional analysis of BC incidence among older US women, we observed differences in BC incidence rates and trends when disaggregating women aged 65 years and older into smaller age groups and stratifying by factors such as race and ethnicity and stage at diagnosis. Notably, distinct trends emerged among the oldest age group (≥85 years), highlighting the importance of evaluating BC incidence in women aged 75 to 84 years and 85 years and older separately from those younger than 75 years. These findings suggest a need to investigate whether age-specific trends are due to guidelines that suggest reduced screening for older age groups. Further research should also explore BC mortality trends in the oldest age groups, considering stage and subtype specific findings. This is particularly important given our finding that racial and ethnic disparities in BC molecular subtypes, which are well-documented in women younger than 65 years, persist in older age groups, alongside a rising incidence of later stage disease. Together, these findings underscore the importance of age- and race and ethnicity–specific analyses to address disparities and improve outcomes in older women.

## Notes

Supplement 1. **eTable 1. **Molecular Subtype Composition of Age-Adjusted In Situ and Invasive Breast Cancer Incidence Rates per 100 000 US Standard Population by Patient Demographic and Diagnosis Characteristics, 2011-2014 vs 2015-2019 **eTable 2. **Annual Percent Changes in Invasive and In Situ Age-Adjusted Breast Cancer Incidence Rates by Geographic, Patient, and Cancer Characteristics, 2001-2019 **eFigure. **Invasive and In Situ Breast Cancer Segmented Trends From 2001-2019, by Race and Ethnicity, Region, and Stage at Diagnosis by Age Group

Supplement 2.
Data Sharing Statement
