# Supplementary material for: Breast Cancer Incidence Trends in Older US Women by Race, Ethnicity, Geography, and Stage
Source: JAMA Netw Open. 2025 Jun 24;8(6):e2516947. doi: 10.1001/jamanetworkopen.2025.16947 (PMC12188358; doi:10.1001/jamanetworkopen.2025.16947)
Supplement: Supplement 1. — eTable 1. Molecular Subtype Composition of Age-Adjusted In Situ and Invasive Breast Cancer Incidence Rates per 100 000 US Standard Population by Patient Demographic and Diagnosis Characteristics, 2011-2014 vs 2015-2019 eTable 2. Annual Percent Changes in Invasive and In Situ Age-Adjusted Breast Cancer Incidence Rates by Geographic, Patient, and Cancer Characteristics, 2001-2019 eFigure. Invasive and In Situ Breast Cancer Segmented Trends From 2001-2019, by Race and Ethnicity, Region, and Stage at Diagnosis by Age Group [file jamanetwopen-e2516947-s001.pdf]

Supplemental Online Content

Lee Argov EJ, Lui ML, Karr AG, Tehranifar P, Kehm RD. Breast cancer incidence trends in older US women by race, ethnicity, geography, and stage. *JAMA Netw Open.* 2025;8(6):e2516947. doi:10.1001/jamanetworkopen.2025.16947

eTable 1. Molecular Subtype Composition of Age-Adjusted In Situ and Invasive Breast Cancer Incidence Rates per 100 000 US Standard Population by Patient Demographic and Diagnosis Characteristics, 2011-2014 vs 2015-2019

eTable 2. Annual Percent Changes in Invasive and In Situ Age-Adjusted Breast Cancer Incidence Rates by Geographic, Patient, and Cancer Characteristics, 2001-2019

eFigure. Invasive and In Situ Breast Cancer Segmented Trends From 2001-2019, With Annual Percent Changes by Race and Ethnicity, Region, and Stage at Diagnosis by Age Group

This supplemental material has been provided by the authors to give readers additional information about their work.

© 2025 Lee Argov EJ et al. *JAMA Network Open*.

eTable 1. Molecular Subtype Composition of Age-Adjusted In Situ and Invasive Breast Cancer Incidence Rates per 100 000 US Standard Population by Patient Demographic and Diagnosis Characteristics, 2011-2014 vs 2015-2019

	**All Ages (65+)**		**Ages 65-74 years**		**Ages 75-84 years**		**Ages 85+**	
	**2011-2014**	**2015-2019**	**2011-2014**	**2015-2019**	**2011-2014**	**2015-2019**	**2011-2014**	**2015-2019**
Overall								
Luminal A	311.5 (310.4-312.6)	323.6 (322.7-324.6)	326.5 (324.9-328.0)	343.6 (342.3-344.9)	322.9 (320.9-324.9)	333.3 (331.5-335.0)	215.1 (212.8-217.4)	210.7 (208.7-212.7)
Luminal B	31.8 (31.5-32.2)	32.1 (31.8-32.4)	35.3 (34.8-35.8)	35.4 (35.0-35.8)	30.5 (29.8-31.1)	31.2 (30.7-31.8)	21.3 (20.5-22.0)	20.9 (20.3-21.6)
ERRB2-enriched	13.2 (13.0-13.4)	12.5 (12.3-12.7)	14.7 (14.4-15.0)	13.5 (13.3-13.8)	12.6 (12.2-13.0)	12.2 (11.8-12.5)	8.6 (8.2-9.1)	9.0 (8.6-9.4)
Triple negative	38.7 (38.3-39.1)	38.7 (38.3-39.0)	41.7 (41.2-42.3)	40.8 (40.3-41.2)	38.2 (37.5-38.9)	39.4 (38.8-40.0)	27.4 (26.6-28.2)	27.7 (27.0-28.4)
Unknown	117.8 (117.1-118.5)	107.6 (107.1-108.2)	125.4 (124.5-126.4)	119.3 (118.5-120.1)	113.7 (112.5-115.0)	100.8 (99.9-101.8)	97.0 (95.4-98.5)	77.6 (76.5-78.8)
Metropolitan Status								
**Metropolitan**								
Luminal A	317.1 (315.8-318.3)	327.0 (326.0-328.1)	331.8 (330.1-333.5)	346.9 (345.4-348.3)	328.8 (326.6-331.1)	337.1 (335.2-339.1)	220.2 (217.7-222.8)	213.4 (211.3-215.6)
Luminal B	31.8 (31.4-32.2)	32.2 (31.9-32.5)	35.3 (34.7-35.9)	35.6 (35.1-36.0)	30.4 (29.7-31.1)	31.1 (30.5-31.7)	21.3 (20.5-22.1)	20.9 (20.3-21.6)
ERRB2-enriched	13.2 (12.9-13.4)	12.5 (12.3-12.8)	14.6 (14.3-15.0)	13.6 (13.3-13.9)	12.6 (12.2-13.0)	12.2 (11.8-12.5)	8.7 (8.2-9.2)	9.1 (8.7-9.6)
Triple negative	39.3 (38.9-39.8)	39.0 (38.6-39.3)	42.3 (41.7-42.9)	41.0 (40.5-41.5)	38.8 (38.0-39.6)	39.7 (39.0-40.3)	28.2 (27.3-29.1)	28.1 (27.3-28.9)
Unknown	120.6 (119.9-121.4)	110.6 (110.0-111.2)	129.5 (128.4-130.6)	123.4 (122.5-124.3)	116.2 (114.9-117.6)	103.2 (102.1-104.3)	95.8 (94.1-97.5)	77.3 (76.0-78.6)
**Non-Metropolitan**								
Luminal A	285.5 (282.9-288.0)	306.3 (304.0-308.5)	301.2 (297.7-304.8)	326.7 (323.6-329.9)	295.4 (290.8-300.0)	314.2 (310.1-318.4)	189.7 (184.5-194.9)	196.1 (191.4-200.8)
Luminal B	31.9 (31.1-32.8)	32.1 (31.4-32.9)	35.1 (33.9-36.4)	34.9 (33.9-35.9)	30.9 (29.5-32.4)	32.0 (30.7-33.3)	21.0 (19.4-22.9)	20.9 (19.4-22.5)
ERRB2-enriched	13.3 (12.8-13.9)	12.2 (11.7-12.6)	14.9 (14.1-15.7)	13.3 (12.6-13.9)	12.8 (11.9-13.8)	11.9 (11.2-12.8)	8.1 (7.1-9.3)	8.2 (7.3-9.3)
Triple negative	36.0 (35.1-36.9)	36.8 (36.0-37.6)	39.2 (37.9-40.5)	39.1 (38.0-40.2)	35.6 (34.0-37.2)	37.5 (36.1-38.9)	23.2 (21.4-25.1)	25.0 (23.4-26.8)
Unknown	104.0 (102.5-105.5)	92.2 (90.9-93.4)	105.8 (103.6-107.9)	97.6 (95.8-99.3)	102.0 (99.3-104.7)	88.9 (86.7-91.2)	102.3 (98.5-106.2)	78.6 (75.6-81.6)
Region								
**Midwest**								
Luminal A	329.4 (327.0-331.9)	344.7 (342.5-346.8)	344.7 (341.3-348.2)	362.4 (359.4-365.3)	343.6 (339.2-348.0)	361.5 (357.6-365.5)	223.5 (218.8-228.4)	220.5 (216.4-224.8)
Luminal B	33.4 (32.6-34.2)	32.4 (31.8-33.1)	37.4 (36.2-38.5)	35.1 (34.2-36.0)	31.5 (30.2-32.9)	32.5 (31.4-33.7)	21.7 (20.3-23.3)	20.7 (19.4-22.0)
ERRB2-enriched	14.0 (13.5-14.5)	12.9 (12.5-13.3)	15.4 (14.6-16.1)	13.7 (13.2-14.3)	13.5 (12.6-14.4)	12.8 (12.1-13.6)	9.5 (8.5-10.5)	9.3 (8.5-10.2)

© 2025 Lee Argov EJ et al. *JAMA Network Open*.

	All Ages (65+)		Ages 65-74 years		Ages 75-84 years		Ages 85+	
	2011-2014	2015-2019	2011-2014	2015-2019	2011-2014	2015-2019	2011-2014	2015-2019
Triple negative	41.4 (40.6-42.3)	41.3 (40.5-42.0)	44.5 (43.2-45.7)	43.3 (42.3-44.3)	41.8 (40.3-43.3)	42.7 (41.4-44.1)	27.4 (25.8-29.1)	28.6 (27.1-30.1)
Unknown	105.7 (104.3-107.1)	101.7 (100.6-102.9)	111.7 (109.7-113.7)	113.6 (112.0-115.3)	103.0 (100.6-105.4)	95.6 (93.5-97.6)	88.0 (85.1-91.1)	69.0 (66.7-71.4)
**Northeast**								
Luminal A	336.2 (333.6-338.6)	343.7 (341.5-346.0(	350.2 (346.5-354.0)	363.2 (360.1-366.4)	355.6 (350.9-360.5)	358.9 (354.7-363.2)	220.4 (215.5-225.4)	216.9 (212.6-221.2)
Luminal B	33.6 (32.8-34.5)	33.2 (32.5-33.9)	36.9 (35.7-38.1)	37.1 (36.1-38.2)	32.9 (31.5-34.4)	31.4 (30.2-32.7)	21.8 (20.3-23.4)	21.4 (20.0-22.8)
ERRB2-enriched	13.1 (12.6-13.6)	12.7 (12.3-13.2)	14.9 (14.2-15.7)	13.9 (13.3-14.5)	11.9 (11.0-12.8)	12.2 (11.5-13.0)	8.7 (7.8-9.8)	9.0 (8.1-9.9)
Triple negative	38.8 (37.9-39.7)	37.9 (37.2-38.7)	41.7 (40.4-43.0)	39.6 (38.6-40.7)	38.5 (36.9-40.1)	38.7 (37.4-40.1)	27.0 (25.3-28.8)	28.1 (26.6-29.7)
Unknown	122.1 (120.5-123.7)	113.0 (111.7-114.3)	131.0 (128.7-133.3)	126.8 (125.0-128.7)	118.5 (115.7-121.3)	106.1 (103.8-108.4)	94.6 (91.4-97.9)	73.9 (71.4-76.5)
**South**								
Luminal A	285.4 (283.7-287.2)	305.0 (303.5-306.5)	301.5 (299.1-303.9)	326.6 (324.5-328.6)	292.1 (289.0-295.3)	310.5 (307.8-313.3)	197.5 (193.7-201.3)	197.4 (194.2-200.7)
Luminal B	31.7 (31.1-32.3)	32.5 (32.0-33.0)	35.1 (34.3-35.9)	35.8 (35.1-36.4)	30.2 (29.2-31.3)	31.8 (31.0-32.7)	21.6 (20.4-22.9)	20.6 (19.6-21.7)
ERRB2-enriched	13.3 (12.9-13.7)	12.6 (12.3-12.9)	14.9 (14.3-15.4)	13.8 (13.4-14.2)	12.9 (12.2-13.6)	12.1 (11.6-12.7)	7.6 (6.9-8.4)	8.6 (8.0-9.4)
Triple negative	38.8 (38.1-39.4)	40.1 (39.6-40.7)	42.4 (41.5-43.4)	43.0 (42.3-43.8)	37.4 (36.3-38.6)	39.9 (38.9-40.9)	27.0 (25.6-28.4)	28.3 (27.1-29.6)
Unknown	123.8 (122.6-124.9)	110.6 (109.7-111.5)	129.4 (127.8-131.0)	120.4 (119.2-121.7)	120.9 (118.8-122.9)	104.3 (102.7-105.9)	108.3 (105.6-111.2)	86.7 (84.6-88.9)
**West**								
Luminal A	317.0 (314.5-319.5)	318.6 (316.6-320.6)	332.4 (329.1-335.8)	339.6 (336.9-342.3)	325.0 (320.5-329.5)	323.3 (319.6-327.1)	228.2 (223.0-233.5)	215.5 (211.1-219.9)
Luminal B	28.9 (28.2-29.6)	30.4 (29.8-31.0)	32.1 (31.1-33.2)	33.7 (32.9-34.6)	27.4 (26.1-28.8)	28.6 (27.5-29.7)	19.5 (18.0-21.1)	21.3 (20.0-22.7)
ERRB2-enriched	12.4 (11.9-12.9)	11.8 (11.4-12.2)	13.5 (12.8-14.2)	12.6 (12.0-13.1)	12.0 (11.1-12.9)	11.5 (10.8-12.2)	9.1 (8.1-10.2)	9.2 (8.3-10.2)
Triple negative	35.9 (35.1-36.7)	34.2 (33.6-34.9)	37.9 (36.7-39.0)	35.4 (34.5-36.3)	35.6 (34.1-37.1)	35.6 (34.4-36.9)	28.3 (26.5-30.2)	25.3 (23.8-26.9)
Unknown	116.1 (114.6-117.6)	104.1 (103.0-105.3)	127.3 (125.2-129.4)	116.6 (115.0-118.2)	108.2 (105.7-110.9)	95.5 (93.5-97.6)	91.2 (88.0-94.6)	75.9 (73.4-78.6)
Race / Ethnicity								
**Hispanic (All Races)**								
Luminal A	214.2 (210.8-217.6)	225.8 (223.0-228.5)	226.7 (222.1-231.3)	251.6 (247.8-255.5)	217.8 (211.8-224.0)	216.9 (212.0-221.9)	150.3 (142.2-158.7)	141.3 (135.2-147.6)
Luminal B	24.8 (23.7-25.9)	24.8 (23.9-25.8)	27.9 (26.3-29.5)	27.9 (26.7-29.2)	23.0 (21.0-25.0)	23.7 (22.1-25.4)	16.8 (14.2-19.8)	15.0 (13.1-17.1)
ERRB2-enriched	9.9 (9.2-10.7)	10.5 (9.9-11.1)	11.4 (10.4-12.5)	12 (11.2-12.9)	9.4 (8.2-10.7)	9.3 (8.3-10.3)	5.3 (3.9-7.1)	7.3 (6.0-8.9)
Triple negative	27.1 (25.9-28.3)	28.2 (27.2-29.2)	28.9 (27.3-30.6)	30.4 (29.1-31.8)	27.1 (25.0-29.4)	27.6 (25.8-29.4)	19.0 (16.2-22.2)	20.5 (18.2-22.9)

© 2025 Lee Argov EJ et al. *JAMA Network Open*.

	All Ages (65+)		Ages 65-74 years		Ages 75-84 years		Ages 85+	
	2011-2014	2015-2019	2011-2014	2015-2019	2011-2014	2015-2019	2011-2014	2015-2019
Unknown	95.9 (93.7-98.2)	89.4 (87.7-91.1)	102.6 (99.6-105.8)	102.6 (100.2-105.0)	90.7 (86.9-94.7)	78.6 (75.6-81.6)	82.1 (76.2-88.4)	64.5 (60.4-68.7)
**Non-Hispanic American Indian /Alaska Native**								
Luminal A	250.5 (236.5-265.1)	241.8 (230.9-253.0)	253.2 (235.6-271.8)	261.7 (247.6-276.5)	271 (244.3-299.7)	245.6 225.4-267.2)	179.7 (144.8-220.3)	145.8 (121.1-174.0)
Luminal B	27.4 (23.0-32.4)	25.8 (22.4-29.6)	32.9 (26.8-40.0)	30.7 (26.1-36.0)	19.3 (12.7-28.1)	22.6 (16.8-29.8)	^	^
ERRB2-enriched	15.3 (12.0-19.2)	11.6 (9.4-14.3)	16.8 (12.5-22.2)	13.4 (10.4-17.0)	15 (9.3-22.9)	11.4 (7.4-16.8)	^	^
Triple negative	28.9 (24.3-34.1)	30.0 (26.3-34.1)	31.6 (25.6-38.7)	32.4 (27.6-37.8)	29.4 (22.7-37.5)	29.4 (22.7-37.5)	^	21.5 (12.7-34.0)
Unknown	95.8 (87.3-104.9)	76.0 (70.1-82.4)	108.1 (96.7-120.6)	85.6 (77.6-94.2)	78.9 (64.9-95.1)	69.3 (58.7-81.2)	91.8 (67.4-122.0)	55 (40.2-73.3)
**Non-Hispanic Asian / Pacific Islander**								
Luminal A	191.7 (187.4-196.1)	202.9 (199.5-206.4)	217.4 (211.4-223.5)	231.9 (227.1-236.8)	178.0 (170.5-185.7)	190.4 (184.3-196.8)	121.8 (112.0-132.1)	115.7 (108.5-123.2)
Luminal B	21.2 (19.8-22.7)	22.3 (21.2-23.5)	25.7 (23.6-27.8)	27.2 (25.5-28.8)	17.8 (15.5-20.4)	18.9 (17.0-21.0)	11.9 (9.0-15.5)	11.7 (9.5-14.3)
ERRB2-enriched	11.4 (10.4-12.5)	10.8 (10.0-11.6)	13.9 (12.4-15.5)	12.5 (11.4-13.6)	9.1 (7.5-11.0)	9.8 (8.4-11.3)	7.4 (5.2-10.4)	6.3 (4.8-8.3)
Triple negative	23.7 (22.2-25.4)	24.2 (23.0-25.4)	25.6 (23.6-27.8)	25.7 (24.1-27.4)	22.2 (19.6-25.0)	24.1 (21.9-26.4)	20.2 (16.4-24.7)	18.0 (15.2-21.1)
Unknown	85.8 (82.9-88.7)	93.3 (91.0-95.7)	103.1 (99.0-107.3)	114.3 111.0-117.8)	74 (69.2-79.1)	79.4 (75.4-83.5)	46.2 (40.2-52.8)	44.1 (39.7-48.8)
**Non-Hispanic Black**								
Luminal A	264.1 (260.8-267.5)	274.1 (271.2-276.9)	272.3 (267.8-276.9)	286 (282.3-289.7)	273.4 (267.2-279.6)	280.4 (275.2-285.7)	202.6 (194.6-210.9)	204.9 (198.2-211.9)
Luminal B	31.2 (30.1-32.4)	32.0 (31.1-33.0)	35.3 (33.6-36.9)	35.7 (34.4-37.0)	28.5 (26.5-30.6)	30.5 (28.8-32.3)	22.2 (19.6-25.0)	21.1 (18.9-23.4)
ERRB2-enriched	15.5 (14.7-16.4)	16.4 (15.7-17.1)	17.4 (16.3-18.6)	17.9 (17.0-18.9)	14.6 (13.2-16.1)	15.8 (14.6-17.1)	10.3 (8.5-12.3)	11.7 (10.1-13.5)
Triple negative	64.3 (62.6-66.0)	68.0 (66.6-69.4)	72.2 (69.9-74.6)	74.0 (72.1-75.9)	60.2 (57.4-63.2)	67.6 (65.0-70.2)	42.2 (38.6-46.1)	43.8 (40.8-47.1)
Unknown	137.1 (134.7-139.6)	128.5 (126.6-130.4)	145.5 (142.2-148.8)	143.7 (141.1-146.4)	131.7 (127.5-136.1)	118.3 (115.0-121.8)	117.0 (110.9-123.3)	93.1 (88.5-97.8)
**Non-Hispanic White**								
Luminal A	331.8 (330.5-333.1)	346.2 (345.0-347.3)	348.6 (346.7-350.5)	367.2 (365.7-368.8)	344.6 (342.2-347.0)	358.5 (356.4-360.6)	223.3 (220.7-225.9)	220.8 (218.6-223.1)
Luminal B	33.1 (32.7-33.5)	33.5 (33.1-33.8)	36.5 (35.9-37.1)	36.6 (36.1-37.1)	32.0 (31.3-32.8)	32.8 (32.2-33.4)	21.8 (21.0-22.6)	21.9 (21.1-22.6)
ERRB2-enriched	13.3 (13.0-13.6)	12.3 (12.0-12.5)	14.6 (14.2-15.0)	13.1 (12.8-13.4)	12.9 (12.5-13.4)	12.1 (11.8-12.5)	8.7 (8.2-9.2)	9.0 (8.5-9.5)
Triple negative	37.5 (37.0-379)	36.8 (36.4-37.1)	40 (39.4-40.6)	38.2 (37.7-38.7)	37.4 (36.7-38.2)	38 (37.3-38.7)	26.8 (25.9-27.7)	27.3 (26.5-28.1)
Unknown	118.4 (117.6-119.2)	106.4 (105.7-107.0)	125.6 (124.4-126.7)	116.6 (115.7-117.5)	115.2 (113.9-116.6)	101 (99.9-102.1)	97.3 (95.6-99.0)	78.3 (76.9-79.7)
Stage at Diagnosis								

© 2025 Lee Argov EJ et al. *JAMA Network Open*.

	All Ages (65+)		Ages 65-74 years		Ages 75-84 years		Ages 85+	
	2011-2014	2015-2019	2011-2014	2015-2019	2011-2014	2015-2019	2011-2014	2015-2019
**In situ**								
Luminal A	7.6 (7.5-7.8)	4.2 (4.1-4.3)	9.1 (8.9-9.4)	4.9 (4.7-5.1)	7.2 (6.9-7.5)	3.9 (3.7-4.1)	2.6 (2.4-2.9)	1.7 (1.5-1.9)
Luminal B	1.9 (1.8-2.0)	0.8 (0.7-0.8)	2.4 (2.3-2.5)	1.0 (0.9-1.0)	1.7 (1.5-1.8)	0.7 (0.6-0.7)	0.5 (0.4-0.7)	0.2 (0.2-0.3)
ERRB2-enriched	0.9 (0.9-1.0)	0.4 (0.4-0.5)	1.1 (1.0-1.2)	0.5 (0.5-0.6)	0.9 (0.8-1.0)	0.4 (0.3-0.5)	0.3 (0.3-0.5)	0.2 (0.1-0.3)
Triple negative	0.8 (0.8-0.9)	0.5 (0.4-0.5)	1.0 (0.9-1.0)	0.5 (0.5-0.6)	0.8 (0.7-0.9)	0.5 (0.4-0.5)	0.4 (0.3-0.5)	0.2 (0.1-0.3)
Unknown	73.8 (73.2-74.3)	75.5 (75.0-76.0)	89.3 (88.5-90.1)	93.7 (93.0-94.4)	68.0 (67.1-69.0)	67.5 (66.7-68.3)	24.3 (23.5-25.0)	21.2 (20.6-21.8)
**Localized**								
Luminal A	221.2 (220.2-222.1)	238.0 (237.1-238.8)	231.2 (229.9-232.5)	254.5 (253.4-255.6)	231.8 (230.1-233.5)	245.8 (244.3-247.3)	147.6 (145.7-149.5)	145.0 (143.3-146.6)
Luminal B	18.2 (17.9-18.5)	19.5 (19.3-19.8)	20.2 (19.8-20.6)	22.0 (21.7-22.4)	17.4 (17.0-17.9)	18.7 (18.2-19.1)	11.8 (11.2-12.3)	11.4 (10.9-11.8)
ERRB2-enriched	6.6 (6.5-6.8)	6.7 (6.6-6.9)	7.4 (7.2-7.6)	7.5 (7.3-7.7)	6.4 (6.1-6.7)	6.5 (6.3-6.8)	4.1 (3.8-4.4)	4.1 (3.8-4.4)
Triple negative	24.4 (24.1-24.8)	24.7 (24.4-24.9)	26.9 (26.5-27.4)	26.9 (26.5-27.2)	24.0 (23.5-24.6)	24.6 (24.1-25.1)	15.1 (14.5-15.7)	15.4 (14.9-16.0)
Unknown	19.5 (19.2-19.7)	12.9 (12.7-13.1)	18.7 (18.3-19.1)	12.7 (12.5-13.0)	20.7 (20.2-21.2)	13.5 (13.1-13.8)	19.1 (18.4-19.8)	12.1 (11.6-12.5)
**Regional**								
Luminal A	66.8 (66.3-67.4)	63.6 (63.2-64.0)	70.9 (70.1-71.6)	67.8 (67.2-68.4)	67.1 (66.2-68.0)	63.9 (63.1-64.7)	49.0 (47.9-50.1)	45.1 (44.1-46.0)
Luminal B	9.0 (8.8-9.2)	8.8 (8.6-8.9)	9.8 (9.5-10.0)	9.3 (9.0-9.5)	8.6 (8.3-9.0)	8.8 (8.5-9.0)	6.7 (6.3-7.2)	6.6 (6.3-7.0)
ERRB2-enriched	4.3 (4.1-4.4)	3.9 (3.8-4.0)	4.7 (4.5-4.8)	4.0 (3.9-4.2)	4.1 (3.9-4.3)	3.8 (3.6-4.0)	3.2 (3.0-3.5)	3.5 (3.3-3.8)
Triple negative	10.5 (10.3-10.7)	10.1 (9.9-10.2)	11.0 (10.7-11.2)	10.2 (9.9-10.4)	10.3 (10.0-10.7)	10.4 (10.1-10.7)	9.1 (8.6-9.5)	8.8 (8.4-9.2)
Unknown	5.7 (5.5-5.8)	3.1 (3.0-3.2)	4.8 (4.6-5.0)	2.6 (2.5-2.7)	5.9 (5.6-6.2)	3.3 (3.1-3.5)	8.6 (8.1-9.0)	4.8 (4.5-5.1)
**Distant**								
Luminal A	13.4 (13.2-13.6)	14.3 (14.1-14.5)	13.4 (13.1-13.8)	13.5 (13.3-13.8)	14.1 (13.7-14.5)	15.9 (15.5-16.3)	11.2 (10.7-11.8)	13.1 (12.6-13.6)
Luminal B	2.5 (2.4-2.6)	2.7 (2.6-2.8)	2.6 (2.5-2.8)	2.8 (2.7-2.9)	2.5 (2.3-2.7)	2.7 (2.6-2.9)	1.7 (1.5-1.9)	2.1 (1.9-2.3)
ERRB2-enriched	1.3 (1.2-1.3)	1.3 (1.2-1.3)	1.4 (1.3-1.5)	1.3 (1.2-1.4)	1.2 (1.1-1.4)	1.3 (1.2-1.4)	0.8 (1.7-1.0)	1.0 (0.9-1.1)
Triple negative	2.7 (2.6-2.8)	3.0 (2.9-3.1)	2.7 (2.5-2.8)	2.8 (2.6-2.9)	2.8 (2.6-3.0)	3.4 (3.2-3.6)	2.3 (2.1-2.6)	2.6 (2.4-2.9)
Unknown	4.9 (4.8-5.1)	4.3 (4.2-4.4)	3.8 (3.6-4.0)	3.1 (2.9-3.2)	5.6 (5.3-5.8)	4.9 (4.6-5.1)	8.1 (7.7-8.6)	7.9 (7.5-8.3)
**Unknown**								
Luminal A	2.5 (2.4-2.6)	3.5 (3.4-3.6)	1.8 (1.7-1.9)	2.9 (2.7-3.0)	2.7 (2.5-2.9)	3.7 (3.6-3.9)	4.6 (4.3-5.0)	5.9 (5.5-6.2)

© 2025 Lee Argov EJ et al. *JAMA Network Open*.

	All Ages (65+)		Ages 65-74 years		Ages 75-84 years		Ages 85+	
	**2011-2014**	**2015-2019**	**2011-2014**	**2015-2019**	**2011-2014**	**2015-2019**	**2011-2014**	**2015-2019**
Luminal B	0.3 (0.3-0.3)	0.4 (0.4-0.4)	0.3 (0.2-0.3)	0.4 (0.3-0.4)	0.3 (0.2-0.3)	0.4 (0.4-0.5)	0.5 (0.4-0.6)	0.7 (0.6-0.8)
ERRB2-enriched	0.1 (0.1-0.1)	0.2 (0.0-0.1)	0.1 (0.1-0.1)	0.2 (0.1-0.2)	0.1 (0.0-0.1)	0.2 (0.1-0.2)	0.1 (0.1-0.2)	0.2 (0.1-0.3)
Triple negative	0.3 (0.3-0.3)	0.5 (0.5-0.5)	0.3 (0.2-0.3)	0.5 (0.4-0.5)	0.3 (0.2-0.4)	0.5 (0.4-0.6)	0.5 (0.4-0.6)	0.7 (0.6-0.8)
Unknown	14.0 (13.7-14.2)	11.8 (11.7-12.0)	8.9 (8.6-9.1)	7.2 (7.1-7.4)	13.5 (13.1-14.0)	11.7 (11.4-12.1)	36.9 (36.0-37.9)	31.7 (31.0-32.5)

Rates include both *in situ* and invasive breast cancers and are age-adjusted to the 2000 US Standard Population.

^ Statistic not displayed due to fewer than 16 cases.

© 2025 Lee Argov EJ et al. *JAMA Network Open*.

eTable 2. Annual Percent Changes in Invasive and In Situ Age-Adjusted Breast Cancer Incidence Rates by Geographic, Patient, and Cancer Characteristics, 2001-2019

	ALL AGES 65+		Ages 65-74 Years		Ages 75-84 Years		Ages 85+ Years	
	**Years**	**APC (95% CI)**	**Years**	**APC (95% CI)**	**Years**	**APC (95% CI)**	**Years**	**APC (95% CI)**
Total US	2001-2005	-1.65 (-2.71, -0.16)	2001-2005	-1.93 (-2.89, -0.66)	2001-2005	-1.10 (-1.90, 0.11)	2001-2009	-0.49 (-0.98, 0.87)
	2005-2010	1.42 (0.64, 2.26)	2005-2010	2.26 (1.43, 2.98)	2005-2019	0.23 (-0.20, 1.10)	2009-2019	-1.57 (-2.72, -1.22)
	2010-2019	0.08 (-0.83, 0.39)	2010-2019	0.39 (-0.17, 0.65)	-	-	-	-
Metropolitan status								
Metropolitan	2001-2005	-1.75 (-2.79, -0.34)	2001-2005	-2.04 (-3.03, -0.74)	2001-2019	0.02 (-0.27, 0.39)	2001-2009	-0.39 (-0.93, 1.22)
	2005-2010	1.52 (0.73, 2.34)	2005-2010	2.38 (1.50, 3.13)	-	-	2009-2019	-1.59 (-2.83, -1.21)
	2010-2019	0.00 (-0.82, 0.29)	2010-2019	0.31 (-0.25, 0.59)	-	-		
Non-Metropolitan	2001-2005	-0.85 (-1.57, 0.31)	2001-2005	-0.76 (-1.68, 0.89)	2001-2005	-1.22 (-1.95, 0.01)	2001-2019	-1.32 (-1.71, -0.94)
	2005-2019	0.52 (0.29, 1.21)	2005-2019	0.94 (0.29, 2.00)	2005-2019	0.39 (0.18, 1.02)	-	-
Region								
Midwest	2001-2005	-1.40 (-2.22, 0.11)	2001-2005	-2.07 (-3.17, -0.54)	2001-2005	-1.55 (-2.24, -0.32)	2001-2019	-1.50 (-1.82, -1.17)
	2005-2019	0.67 (0.44, 1.33)	2005-2011	2.05 (1.43, 3.14)	2005-2019	0.56 (0.37, 0.90)	-	-
	-	-	2011-2019	0.47 (-0.38, 0.83)	-	-	-	-
Northeast	2001-2019	0.43 (0.23, 0.65)	2001-2014	1.12 (0.85, 2.40)	2001-2019	0.25 (0.01, 0.48)	2001-2019	-1.21 (-1.54, -0.88)
	-	-	2014-2019	-0.22 (-1.67, 0.72)	-	-	-	-
South	2001-2005	-1.63 (-2.53, -0.43)	2001-2005	-2.06 (-3.22, -0.42)	2001-2005	-1.17 (-2.03, 0.20)	2001-2009	0.38 (-0.22, 1.79)
	2005-2010	1.55 (0.88, 2.24)	2005-2010	2.37 (1.48, 3.28)	2005-2019	0.37 (0.02, 1.29)	2009-2019	-1.48 (-2.29, -1.06)
	2010-2019	0.30 (-0.40, 0.55)	2010-2019	0.65 (-0.16, 0.95)	-	-	-	-
West	2001-2005	-2.69 (-4.07, -0.77)	2001-2005	-2.92 (-4.08, -1.41)	2001-2005	-2.19 (-3.26, -0.59)	2001-2019	-1.15 (-1.59, -0.69)
	2005-2010	1.36 (0.35, 2.47)	2005-2010	2.26 (1.23, 3.11)	2005-2019	-0.18 (-0.49, 0.80)	-	-
	2010-2019	-0.37 (-1.55, 0.05)	2010-2019	-0.09 (-0.74, 0.24)	-	-	-	-
Race / Ethnicity								
Hispanic	2001-2019	0.57 (0.34, 0.85)	2001-2019	1.36 (1.16, 1.59)	2001-2019	-0.16 (-0.53, 0.27)	2001-2019	-1.33 (-1.87, -0.67)

© 2025 Lee Argov EJ et al. *JAMA Network Open*.

	ALL AGES 65+		Ages 65-74 Years		Ages 75-84 Years		Ages 85+ Years	
	**Years**	**APC (95% CI)**	**Years**	**APC (95% CI)**	**Years**	**APC (95% CI)**	**Years**	**APC (95% CI)**
Non-Hispanic American Indian /Alaska Native	2001-2013	2.48 (1.63, 4.95)	2001-2011	3.98 (2.32, 10.97)	2001-2014	1.12 (0.38, 5.25)	2001-2014	2.47 (0.65, 13.36)
	2013-2019	-2.19 (-5.59, -0.08)	2011-2019	-0.93 (-5.13, 0.98)	2014-2019	-2.57 (-6.15, -0.02)	2014-2019	-7.37 (-17.51, -0.94)
Non-Hispanic Asian / Pacific Islander	2001-2019	1.54 (1.17, 2.01)	2001-2019	2.19 (1.85, 2.66)	2001-2019	0.89 (0.52, 1.34)	2001-2019	-0.71 (-1.61, 0.47)
Non-Hispanic Black	2001-2011	1.95 (1.53, 3.03)	2001-2012	2.35 (1.99, 3.00)	2001-2009	1.90 (1.27, 3.69)	2001-2009	1.13 (0.18, 3.29)
	2011-2019	0.28 (-0.81, 0.84)	2012-2019	0.49 (-0.43, 1.08)	2009-2019	0.35 (-0.68, 0.77)	2009-2019	-1.38 (-2.65, -0.78)
Non-Hispanic White	2001-2005	-1.93 (-2.84, -0.72)	2001-2005	-2.35 (-3.34, -1.04)	2001-2005	-1.15 (-1.97, 0.16)	2001-2019	-1.04 (-1.36, -0.73)
	2005-2010	1.47 (0.74, 2.18)	2005-2010	2.27 (1.39, 3.03)	2005-2019	0.32 (0.04, 1.26)	-	-
	2010-2019	0.05 (-0.69, 0.32)	2010-2019	0.25 (-0.35, 0.54)	-	-	-	-
Stage at Diagnosis								
In Situ	2001-2009	1.47 (0.72, 3.29)	2001-2009	1.69 (0.90, 4.12)	2001-2009	1.21 (0.37, 2.90)	2001-2009	1.13 (0.12, 2.74)
	2009-2019	-0.78 (-1.72, -0.26)	2009-2019	-0.16 (-1.56, 0.34)	2009-2019	-1.45 (-2.48, -0.86)	2009-2015	-6.19 (-8.13, -4.66)
	-	-	-	-	-	-	2015-2019	-1.17 (-4.13, 1.11)
Localized	2001-2005	-2.37 (-3.27, -1.16)	2001-2005	-2.72 (-3.74, -1.34)	2001-2005	-1.91 (-2.81, 0.-05)	2001-2019	-0.66 (-0.94, -0.36)
	2005-2011	2.13* (1.61, 2.97)	2005-2011	3.01 (2.38, 3.97)	2005-2019	1.04 (0.76, 1.77)	-	-
	2011-2019	0.92 (0.23, 1.21)	2011-2019	1.29 (0.65, 1.62)	-	-	-	-
Regional	2001-2008	0.38 (-0.35, 1.85)	2001-2009	0.25 (-0.39, 1.62)	2001-2008	0.12 (-0.62, 1.81)	2001-2008	1.43 (0.41, 3.22)
	2008-2019	--1.60 (-2.24, -1.24)	2009-2019	-1.63 (-2.41, -1.20)	2008-2019	-1.57 (-2.46, -1.20)	2008-2019	-2.34 (-3.08, -1.83)
Distant	2001-2014	1.79 (1.58, 2.29)	2001-2013	2.12 (1.82, 2.50)	2001-2019	1.64 (1.33, 1.96)	2001-2019	1.64 (1.36, 1.95)
	2014-2019	0.17 (-0.83, 1.03)	2013-2019	-0.83 (-1.86, -0.11)	-	-	-	-
Unknown	2001-2006	-9.43 (-13.07, -5.63)	2001-2006	-11.37 (-15.21, -8.39)	2001-2006	-9.30 (-12.13, -6.85)	2001-2006	-6.38 (-9.01, -3.78)
	2006-2019	-1.69 (-2.56, 0.51)	2006-2019	-0.99 (-1.88, 0.13)	2006-2015	-2.95 (-5.71, -0.83)	2006-2019	-2.80 (-3.70, 0.04)
	-	-	-	-	2015-2019	2.30 (-1.70, 5.25)	-	-

'Unknown' comprises unknown, unstaged, unspecified, and death certificate only cases.

© 2025 Lee Argov EJ et al. *JAMA Network Open*.

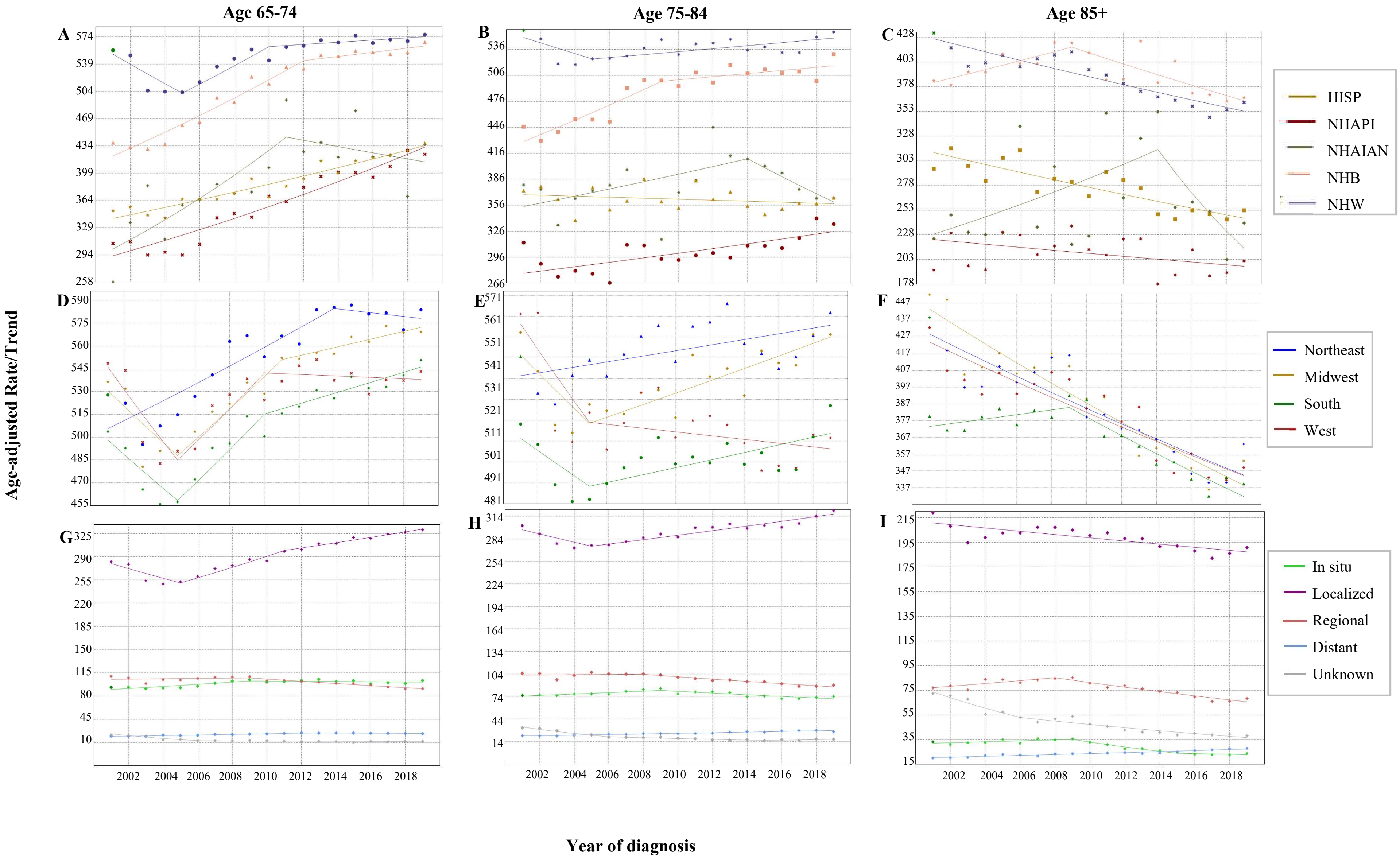

eFigure. Invasive and In Situ Breast Cancer Segmented Trends From 2001-2019, With Annual Percent Changes by Race and Ethnicity, Region, and Stage at Diagnosis by Age Group

Rates are per 100,000 and age-adjusted to the 2000 US Standard Population (19 age groups - Census P25-1130). Figures represent the best fitting model using Joinpoint regression allowing for a maximum of 3 joinpoints and required a minimum of 4 years within each segment and between each segment and either end. Due to COVID-19 related declines in incidence influencing the trend, 2020 was excluded from the Joinpoint regression trendline and calculations.

© 2025 Lee Argov EJ et al. *JAMA Network Open*.
