# Supplementary material for: Breast Cancer Incidence Trends in Older US Women by Race, Ethnicity, Geography, and Stage
Source: JAMA Netw Open. 2025 Jun 24;8(6):e2516947. doi: 10.1001/jamanetworkopen.2025.16947 (PMC12188358; doi:10.1001/jamanetworkopen.2025.16947)
Supplement: Supplement 2. — Data Sharing Statement [file jamanetwopen-e2516947-s002.pdf]

Data Sharing Statement

Lee Argov. Breast Cancer Incidence Trends in Older US Women by Race, Ethnicity, Geography, and Stage. *JAMA Netw Open*. Published June 24, 2025. doi:10.1001/jamanetworkopen.2025.16947

Data
Data available: Yes
Data types: Deidentified participant data
How to access data: Data are publicly available: United States Cancer Statistics public use database https://www.cdc.gov/united-states-cancer-statistics/public-use/index.html, available through SEER*Stat https://seer.cancer.gov/data/access.html
When available: With publication

Supporting Documents
Document types: None

Additional Information
Who can access the data: Data are available for public use in accordance with the United States Cancer Statistics database.
Types of analyses: Data are available for public use in accordance with the United States Cancer Statistics database.
Mechanisms of data availability: Information on data availability can be found here from the United States Cancer Statistics public use database website (https://www.cdc.gov/united-states-cancer-statistics/public-use/index.html).
